# Drinking Natural Mineral Water Maintains Bone Health in Young Rats With Metabolic Acidosis

**DOI:** 10.3389/fnut.2022.813202

**Published:** 2022-03-21

**Authors:** Yao Tan, Anwei Xu, Zhiqun Qiu, Lingqiao Wang, Jia Wang, Jiaohua Luo, Hui Zeng, Huidong Jin, Yushu Wang, Jing Xue, Yujing Huang, Weiqun Shu

**Affiliations:** ^1^Department of Environmental Hygiene, College of Preventive Medicine, Army Medical University (Third Military Medical University), Chongqing, China; ^2^Department of Anti-epidemic, Navy Hospital, Dalian, China; ^3^Department of Emergency, First Hospital Affiliated to Army Medical University (Southwest Hospital), Chongqing, China; ^4^Biomedical Analysis Center, Army Medical University (Third Military Medical University), Chongqing, China

**Keywords:** natural mineral drinking water, bicarbonate, metabolic acidosis, bone mineral density, bone microstructure

## Abstract

**Introduction:**

Metabolic acidosis affects bone health. It remains unclear whether drinking natural mineral water is better for maintaining bone health in the youth with metabolic acidosis.

**Materials and Methods:**

Sixty young female rats (3-weeks-old) were randomly divided into three groups and drank purified water (PW, as control), bicarbonate-rich natural mineral water (Bic-NMW), or sulfate-rich natural mineral water (Sul-NMW), which, respectively, contained calcium (0.17, 155, and 175 mg/L), bicarbonate (0.1360, and 139 mg/L) and sulfate (0, 35.6, and 532 mg/L), for 16 weeks. In the last 3 weeks, metabolic acidosis was induced in 10 rats per group by adding NH_4_Cl (0.28 mM) to drinking water. The rats' blood, urine, and femur were collected for assessing acid-base status, calcium metabolism, bone microstructure, and strength. The difference between the three groups was determined using one-way ANOVA followed by the Student–Newman–Keuls test or Dunnett's T3 test.

**Results:**

Compared with the PW rats, the Bic-NMW rats and the Sul-NMW rats had less urine net acid excretion (−1.51, 0.20 vs. 10.77, EQ/L), higher bone mineral density (442.50, 407.49 vs. 373.28, mg/mm^3^), growth cartilage width (271.83, 283.83 vs. 233.27, μm) and cortical trabecular area (9.33, 9.55 vs. 5.05, mm^2^), and smaller cortical marrow cavity area (5.40, 5.49 vs. 7.27, mm^2^) in the femur (*P* < 0.05). Besides, the Bic-NMW rats had less serum calcium (2.53 vs. 2.68, mmol/L) and C-terminal cross-linked telopeptide of type-I collagen (1.35 vs. 1.93, ng/mL), and higher serum calcitonin (0.61 vs. 0.39, μg/L), femoral trabecular thickness (0.10 vs. 0.09, μm), bone volume/total volume (0.42 vs. 0.34, %), cortical bone area (15.91 vs. 12.80, mm^2^), and ultimate stress (35.12 vs. 29.32, MPa) (*P* < 0.05). The Sul-NMW rats had more osteoclasts (22.50 vs. 11.54, cells/field) (*P* < 0.05).

**Conclusions:**

Drinking natural mineral water, especially bicarbonate-rich natural mineral water, is effective in improving bone health in young rats with metabolic acidosis. These benefits include maintaining bone mineral density, and improving bone microstructure and biomechanical properties *via* moderating metabolic acidosis.

## Introduction

Acidosis is a pathologic condition of acid accumulation or depletion of the base in body. Occidental diet, which is high in acidogenic foods like meat and dairy products and deficient in vegetables, is a classical cause for metabolic acidosis (1, 2). It is popularly adopted in Chinese, resulting in widespread metabolic acidosis in young Chinese.

Under acidosis, the body will neutralize excess acids to achieve acid-base homeostasis through two compensating systems. First, hydrogen ions (H^+^) in the blood can be neutralized by bicarbonate (${\text{HCO}}_{3}^{-}$) and be excreted as carbon dioxide by the respiratory system. These processes decrease the partial pressure of carbon dioxide (PCO_2_), base-excess (BE), and bicarbonate in the blood. Second, hydrogen ions in the blood can be excreted *via* the urinary system as hydrogen ions and ammonium ions (${\text{NH}}_{4}^{+}$). ${\text{HCO}}_{3}^{-}$ in the blood can be increased by elevating its resorption in the kidney. This process increases the titrated acid (TA) and ${\text{NH}}_{4}^{+}$, and decreases ${\text{HCO}}_{3}^{-}$ in urine. Compensation of acidosis through the renal system needs other alkaline ions to buffer the hydrogen ion in blood. Calcium from bone is an essential buffer in this progress (3, 4). Endogenous and exogenous acids stimulate bone resorption to release calcium and other alkalis into the blood to maintain the body's acid-base balance (5–10). This procedure often disturbs the calcium regulating system, and induces excessive bone resorption and blood calcium increasing, resulting in destroying bone microstructure, reducing bone minerals, and weakening bone biomechanical properties (5, 9–11).

Drinking water with specific natural minerals, such as calcium and bicarbonate, can benefit bone health and prevent osteoporosis (12–18). Our previous studies showed that drinking mineral-rich water could improve bone mechanical properties, bone mineral content, and bone development in rats (19). Several researchers found that the natural mineral water exerts more good effects on the trabecular number, bone surface density, and bone volumetric fraction in ovariectomized female Sprague Dawley rats (20). Furthermore, minerals in water are lower but can be more easily absorbed by the body due to its ionic species compared with mineral supplements which are used to treat serious acidosis in the clinic. Mineral water can stably, constantly, and effectively supply minerals, including calcium and bicarbonate (21, 22). It can mildly relieve body acid load, continually maintain body acid-base balance (18), and eventually promote bone health (22). Many studies suggested that mineral water, especially with high calcium and bicarbonate concentrations, is an ideal alternative to medicinal alkaline salts for preventing osteoporosis in the elderly population under chronic acid load (22, 23). However, no study has evaluated the effect of minerals and alkalis in drinking natural water on bone health in youth with acidosis of dietary acid load scores.

To investigate the effect of drinking natural mineral water on bone health in the young individuals with metabolic acidosis, we established an animal model to assess if drinking natural mineral water, especially the bicarbonate-rich natural mineral water, can lessen acid-load, and improve the bone microstructure and biomechanical properties in rats with metabolic acidosis.

## Materials and Methods

### Drinking Water

Three types of bottled water with different mineral content were used in this study: purified water (PW), bicarbonate-rich natural mineral water (Bic-NMW), and sulfate-rich natural mineral water (Sul-NMW). Their pH, total dissolved solids (TDS), total hardness (TH), sodium (Na^+^), potassium (K^+^), calcium (Ca^2+^), magnesium (Mg^2+^), sulfate (${\text{SO}}_{4}^{2-}$), chloride (Cl^−^), ${\text{HCO}}_{3}^{-}$, phosphorus (P), and metasilicate acid (H_2_SiO_3_) were tested by Chongqing Geological and Mineral Testing Center according to the standard examination methods for drinking water (GB/T5750-2006, China), national food safety standards for packaged drinking water (GB 19298-2014, China), and methods for examination of natural mineral drinking water (GB/T8538-2008, China).

### Animal Model

According to a protocol approved by the Institutional Animal Use and Care Committee of Army Medical University (Chongqing, China), all animal procedures were performed and were carried out by individuals with appropriate licenses. Sixty female Sprague-Dawley rats (postnatal 3 weeks and weighing 50.53 ± 7.15 g) were obtained from the Experimental Animal Center of Army Military Medical University (license: SCXK2012-0003). The rats were fed with the standard breeding food (Laboratory Feeds, TMMU, China license: SCX2012-0009), were housed in polypropylene cages with dust-free wood husks as bedding materials, and were kept in a well-ventilated room maintained of 50–60% humidity at 23–25°C with 12 h light and 12 h dark cycles. The body weight, food, and water consumption of the rats were assessed every week.

After 1 week of adaptation, sixty rats were randomly divided into three groups (20 per group) and supplied with the three types of drinking water for 13 weeks. Ten rats from each group were euthanized by 10% chloral hydrate (intraperitoneal injections, 3 mL/kg body weight) after 12 h of fasting. The abdominal aortic blood was collected for assessing blood pH value. The heart blood was collected for determining serum calcium and calcium regulatory hormones. The femur without soft tissue was collected to evaluate bone microstructure.

The left 10 rats per group were induced metabolism acidosis by intaking NH_4_Cl (0.28 mM in drinking water every day) for 3 weeks (these 30 rats had been studied for 16 weeks all told) (24). The rats were then fasted for 12 h, and the rats' urine was collected using metabolic cages during the fasting. After it, these rats were euthanized by 10% chloral hydrate. The abdominal aortic blood was collected for assessing acid-base status with blood gas analysis (25). The heart blood was collected to evaluate serum calcium and magnesium, calcium regulatory hormones, and bone modeling. The femur without soft tissue was collected for assessing bone histopathological, microstructural, and biomechanical properties.

### Acid–Base Status Analysis

The pH value, BE, standard bicarbonate radical (${\text{HCO}}_{3\text{std}}^{-}$), PCO_2_, actual ${\text{HCO}}_{3}^{-}$, and total carbonic acid (TCO_2_) were tested by an automatic blood gas analyzer (ORION-II, Medical Measurement Systems B.V., Holland). The metabolic acidosis was identified when pH was <7.30 (26, 27).

### Renal Net Acid Excretion Analysis

${\text{NH}}_{4}^{+}$, TA, and ${\text{HCO}}_{3}^{-}$ in urine were measured according to the method of Lüthy et al. (28). Creatinine in urine was assessed by commercial enzyme-linked immunosorbent assay (ELISA) kits (creatinine: ml611014, Shanghai Enzyme-linked Biotechnology Co., Ltd., Shanghai, China) according to their procedure. Net acid excretion (NAE) from the kidney was calculated using the formula: NAE = ${\text{NH}}_{4}^{+}$ + TA–${\text{HCO}}_{3}^{-}$ (29).

### Calcium and Magnesium Metabolism, Calcium Regulatory Hormones, and Biomarkers of Bone Modeling

The concentrations of serum total calcium, serum calcium ion (Ca^2+^), serum Mg^2+^, serum albumin (ALB), urine Ca^2+^, and urine Mg^2+^ were assessed with clinical automatic biochemical analysis (UniCel DxC 800 Synchron Clinical Systems, Beckman Coulter, USA). 25-hydroxy vitamin D [25 (OH) vitamin D], calcitonin, parathyroid hormone (PTH), and bone modeling biomarkers including bone alkaline phosphate (BALP, which represented the activity of osteoblast), procollagen type I N-terminal propeptide (PINP, which meant the progress of bone formation), and C-terminal cross-linked telopeptide of type-I collagen (CTx, which represented the bone resorption) in heart blood were assessed by using commercial enzyme-linked immunosorbent assay (ELISA) kits (25 (OH) vitamin D: ml028285; calcitonin: ml002886; PTH: ml002989; BALP: ml003415; PINP: ml038224; CTx: ml028330, Shanghai Enzyme-linked Biotechnology Co., Ltd., Shanghai, China) according to their procedure.

### Bone Microstructure Analysis and Histomorphometric Study

The left distal femur was scanned by the three-dimensional microcomputed tomography (micro-CT) system (VivaCT 40, SCANCO Medical AG, Switzerland). Three-dimensional image data were reconstructed in the metaphysis, defined as a region from 2.0 to 4.0 mm beneath the growth plate. Bone mineral density (BMD), bone volume/total volume (BV/TV), bone surface/bone volume (BS/BV), trabecular thickness (Tb.Th), trabecular bone number (Tb.N), and trabecular separation (Tb. Sp) were calculated according to the guidelines established by the American Society for Bone and Mineral Research Histomorphometry Nomenclature Committee (ASBMR) (30).

The right femur was used for histomorphometric measurement and counting osteoclasts as described by ASBMR (30). Briefly, the right femur was decalcified, stained with hematoxylin and eosin, and scanned in a Virtual Slide System using a whole-slide scanner (40 ×, VS120, Olympus Corporation, Tokyo, Japan). Cortical bone parameters (cortical bone area, cortical trabecular area, and cortical marrow cavity area) and cancellous bone parameters (cancellous bone area, cancellous trabecular area, and cancellous marrow cavity area) were measured in the two μm region below the lowest bony line of the cancellous subperiosteal bone collar area. Bone growth cartilage width, proliferative zone width, and hypertrophic cartilage width were quantified in the bone plate at six equidistant points (31). Osteoclasts were stained by Tartrate-resistant acid phosphatase (TRAP) staining kit (Servicebio, G1050). TRAP positive cells with three or more nuclei were identified as osteoclasts. Osteoclasts number (cells/filed) were determined in six fields (40 ×) of each slide.

### Bone Biomechanical Testing

After the micro-CT examination, the biomechanical properties of the left femur bone were estimated by a three-point bending test (32). The bone was placed horizontally on two supporting bars with a 40 mm span, and the central loading applied force at mid-diaphysis. As previously described (32), the femur was then loaded with a tensile loading speed of 2 mm/s until fracture. The ultimate load (N), maximum deflection (mm), young modulus (MPa), ultimate stress (MPa), and ultimate strain (mm/mm) were detected with a universal material testing machine (Instron1011, Instron Corporation, USA).

### Statistical Analysis

All statistical analyses were performed with SPSS software (SPSS Inc. Released in 2009. PASW Statistics for Windows, Version 18.0. Chicago: SPSS Inc., IL, USA). Data were presented as means ± SEM. Normal distribution was confirmed in all tests performed by one-sample Kolmogorov–Smirnov Test. One-way ANOVA followed by the Student–Newman–Keuls test (homogeneity of variance) and Dunnett's T3 test (heterogeneity of variance) was used to compare differences in three groups on body weight, diet and water intake, serum and urine calcium and magnesium, serum calcium regulatory hormones, biomarkers of acid-base balance and bone modeling, and parameters of bone structure and biomechanics. Independent sample *T*-test was conducted to measure the change of water consumption between the adjacent 2 weeks. *P* < 0.05 was viewed of statistical significance.

## Results

### Minerals in Water

The purified water was low in minerals and alkalis (Table 1). The bicarbonate-rich natural mineral water had similar calcium concentrations (89%) to sulfate-rich natural mineral water (Table 1). The bicarbonate-rich natural mineral water had more bicarbonate (nearly ten times than sulfate-rich natural mineral water) (Table 1). And the sulfate-rich natural mineral water had more sulfate (more than 10 times than bicarbonate-rich natural mineral water) (Table 1).

**Table 1:** Minerals in three types of drinking water.

	**PW**	**Bic-NMW**	**Sul-NMW**
pH value	6.33	7.50	8.21
Total dissolved solids (mg/L)	3.63	1,934.23	953.79
Total hardness (CaCO_3_,mg/L)	0.43	766.21	650.67
Calcium (mg/L)	0.17	155.33	175.15
Magnesium (mg/L)	0.08	94.76	54.65
Sodium (mg/L)	0.33	174.40	3.89
Potassium (mg/L)	0.04	11.97	7.31
Sulfates (mg/L)	<0.0002	35.64	531.79
Chlorides (mg/L)	<0.10	58.38	10.03
Bicarbonate (mg/L)	3.09	1,360.70	139.16
Phosphorus (mg/L)	0.03	0.05	0.03
Silicic acid (mg/L)	<0.10	40.81	22.74

*PW, purified water; Bic-NMW, the bicarbonate-rich mineral water; Sul-NMW, the sulfate-rich mineral water (33)*.

### Bodyweight, Diet, and Water Consumption

There were no significant differences among the three groups in body weight, diet, and water intake during 16 weeks of water drinking (Supplementary Figures 1A–C). In all groups, the water consumption significantly decreased at the 14th week compared with that at the 13th week [13th vs. 14th (mean ± SEM, *n* = 10): PW, 32.50 ± 0.37 vs. 22.84 ± 0.03, *P* < 0.05; Bic-NMW, 32.12 ± 0.25 vs. 22.77 ± 0.17, *P* < 0.05; Sul-NMW, 31.79 ± 0.46 vs. 22.79 ± 0.02, *P* < 0.05], which might result from the change of water taste due to the addition of NH_4_Cl into drinking water. The water consumption did not significantly differ between the other 2 weeks in a row in each group.

### Comparison of Acid-Base Balance After the Acid Load

**Figure 1:**
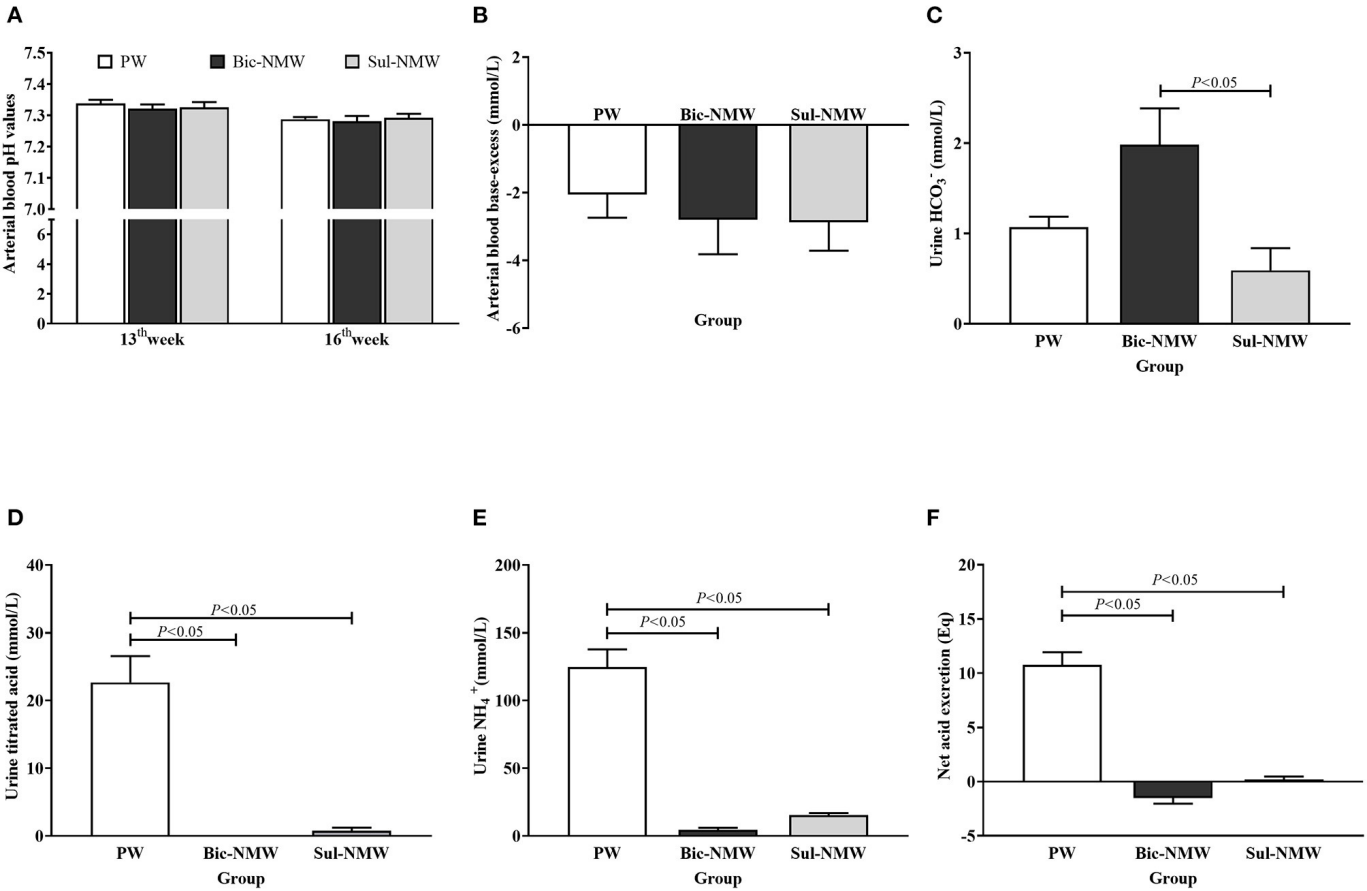
Acid-base balance in young rats **(A–F)**. **(A)** Arterial blood pH values of rats before (13th week) and after (16th week) the acid load. **(B)** Arterial blood base excess of rats with the acid load (16th week). **(C)** Urine bicarbonate ion (${\text{HCO}}_{3}^{-}$) concentration of rats with the acid load (16th week). **(D)** Urine titrated acid of rats with the acid load (16th week). **(E)** Urine ammonium ion (${\text{NH}}_{4}^{+}$) concentration of rats with the acid load (16th week). **(F)** Net acid excretion of rats with the acid load (16th week). The values are presented as means with error bars indicating SEM; *n* = 10 rats/group. PW, the purified water group; Bic-NMW, the bicarbonate-rich mineral water group; Sul-NMW, the sulfate-rich mineral water group.

Before acidosis was initiated, the blood pH values in the three groups were above 7.30 and did not have a significant difference at the 13th week (Figure 1A). After the acid load was induced, the blood pH values in the three groups were below 7.30, and BE was negative at the 16th week (Figures 1A,B). There was no significant difference in blood pH and BE among the three groups (*P* ≥ 0.05, Figures 1A,B). The standard bicarbonate (${\text{HCO}}_{3\text{std}}^{-}$), PCO_2_, actual ${\text{HCO}}_{3}^{-}$, TCO_2_, and the ratio of actual bicarbonate radical to total carbonic acid (${\text{HCO}}_{3}^{-}$/TCO_2_) in blood did not have a significant difference among the three groups (*P* ≥ 0.05, Supplementary Figures 2A–E).

The Bic-NMW group had significantly higher urine ${\text{HCO}}_{3}^{-}$ than the Sul-NMW group (*P* < 0.05, Figure 1C). The two mineral water groups (both the Bic-NMW group and Sul-NMW group) had significantly higher urine TA, ${\text{NH}}_{4}^{+}$, and NAE than the PW group (*P* < 0.05, Figures 1D–F).

### Comparison of Calcium and Magnesium Metabolism, Calcium Regulatory Hormones, and Bone Modeling Markers After the Acid Load

**Figure 2:**
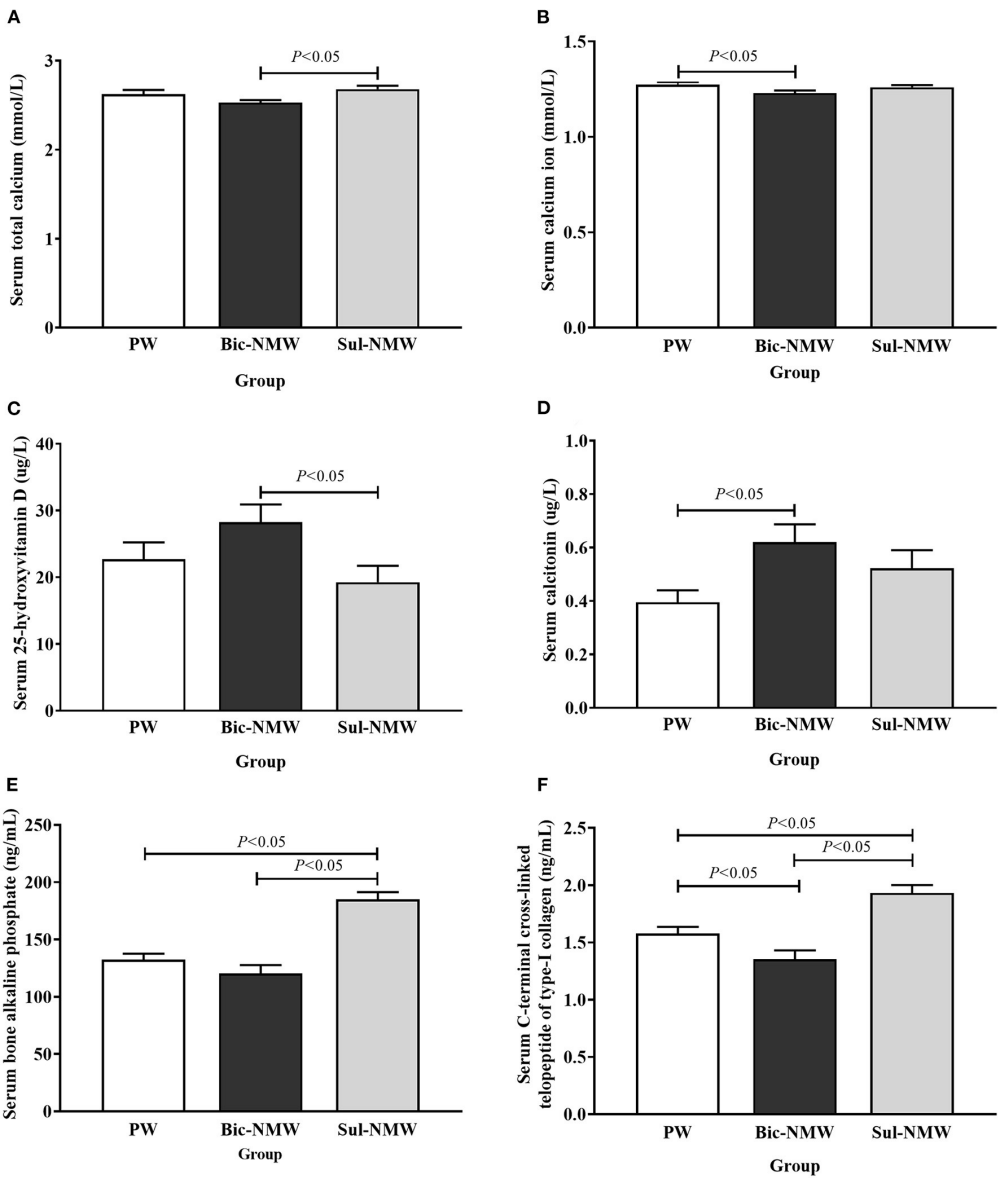
Blood calcium **(A,B)**, calcium regulatory hormones **(C,D)**, and bone modeling markers **(E,F)** of young rats with the acid load (16th week). **(A)** Serum total calcium concentration. **(B)** Serum calcium ion concentration. **(C)** Serum 25-hydroxyvitamin D concentration. **(D)** Serum calcitonin concentration. **(E)** Serum bone alkaline phosphate concentration. **(F)** Serum C-terminal cross-linked telopeptide of type-I collagen concentration. The values are presented as means with error bars indicating SEM; *n* = 10 rats/group. PW, the purified water group; Bic-NMW, the bicarbonate-rich mineral water group; Sul-NMW, the sulfate-rich mineral water group.

The Bic-NMW group had significantly lower serum total calcium than the Sul-NMW group and significantly lower serum Ca^2+^ than the PW group (*P* < 0.05, Figures 2A,B). The serum ALB, serum Mg^2+^, urine Mg^2+^, and urine Ca^2+^ had no significant difference among the three groups (*P* ≥ 0.05, Supplementary Figures 2F, 3A–C). The Bic-NMW group had significantly higher serum 25 (OH) vitamin D levels than the Sul-NMW group and significantly higher serum calcitonin than the PW group (*P* < 0.05, Figures 2C,D). The serum PTH had no significant difference among the three groups (*P* ≥ 0.05, Supplementary Figure 3D). The serum BALP was significantly higher in the Sul-NMW group than in the PW and Bic-NMW groups (*P* < 0.05, Figure 2E). The serum PINP had no significant difference among the three groups (*P* ≥ 0.05, Supplementary Figure 3E). The CTx was significantly higher in the Sul-NMW group than that in the PW group and the Bic-NMW group, and was lower in the Bic-NMW group than that in the PW group (*P* < 0.05, Figure 2F). The serum Ca^2+^, serum 25 (OH) vitamin D, and serum calcitonin were not significantly different among the three groups before acidosis was initiated (at the 13th week, *P* ≥ 0.05, Supplementary Figures 4A–C).

### Comparison of Bone Microstructures After the Acid Load

**Figure 3:**
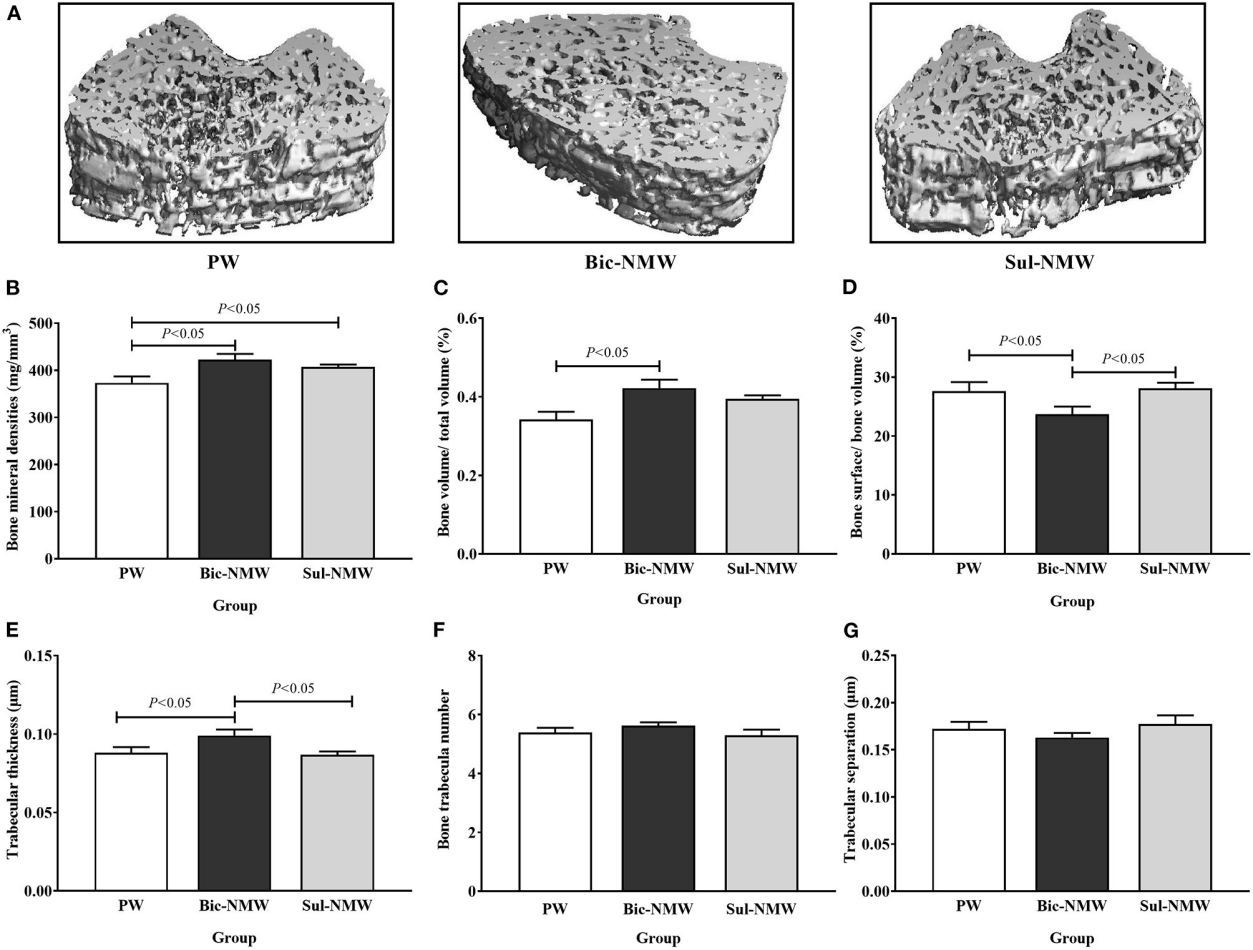
Pathological changes of femora of young rats with the acid load (16th week). **(A)** Representative three-dimensional microcomputed tomography images of femoral bones by micro-CT. **(B)** Bone mineral density. **(C)** Bone volume/total volume fraction. **(D)** Bone surface/bone volume fraction. **(E)** Trabecular thickness. **(F)** Bone trabecula number. **(G)** Trabecular separation. The values are presented as means with error bars indicating SEM; *n* = 10 rats/group. PW, the purified water group; Bic-NMW, the bicarbonate-rich mineral water group; Sul-NMW, the sulfate-rich mineral water group.

Compared with the PW group, the two mineral water groups had significantly higher bone mineral density levels (*P* < 0.05, Figures 3A,B), and the Bic-NMW group had significantly higher BV/TV (*P* < 0.05, Figure 3C). Besides, the Bic-NMW group had significantly lower bone surface/bone volume and greater trabecular thickness than the other two groups (all *P* < 0.05, Figures 3D,E). No significant difference in the trabecular bone number and trabecular separation was found among the three groups (*P* ≥ 0.05, Figures 3F,G). There were no significant differences among the three groups in all bone microstructure parameters at the 13th week (*P* ≥ 0.05, Supplementary Figures 5A–F).

### Comparison of Histopathological and Histomorphometric Characteristics of the Bone After the Acid Load

**Figure 4:**
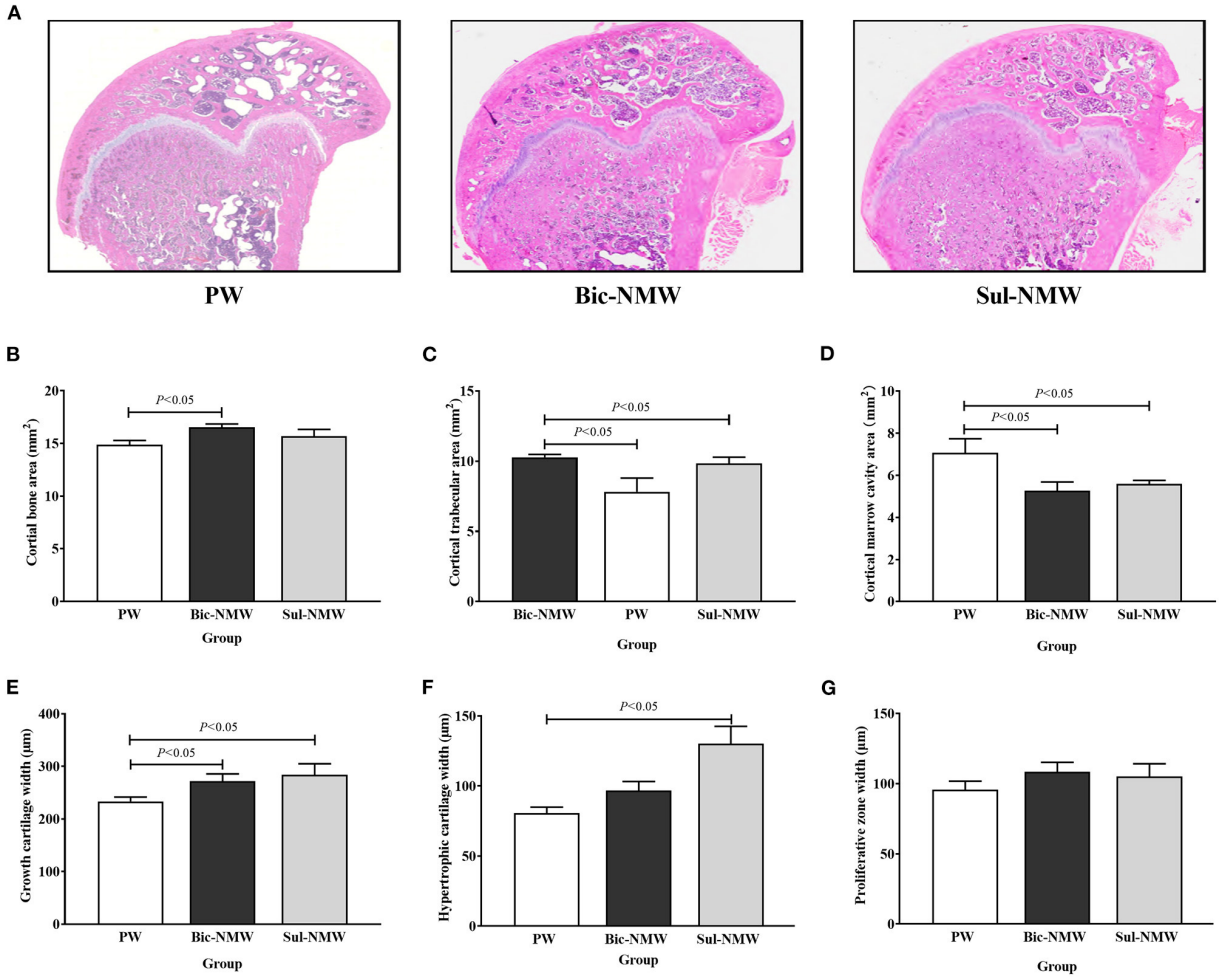
Histopathological changes of femora of young rats with the acid load (16th week). **(A)** Representative full slide images of femora by hematoxylin and eosin staining. **(B)** Cortical bone area. **(C)** Cortical trabecular area. **(D)** Cancellous marrow cavity area. **(E)** Growth cartilage width. **(F)** Hypertrophic cartilage width. **(G)** Proliferative zone width. **(H)** cancellous bone area. **(I)** Cancellous trabecular area. **(J)** Cancellous bone marrow cavity area. The values are presented as means with error bars indicating SEM; *n* = 10 rats/group. PW, the purified water group; Bic-NMW, the bicarbonate-rich mineral water group; Sul-NMW, the sulfate-rich mineral water group.

**Figure 5:**
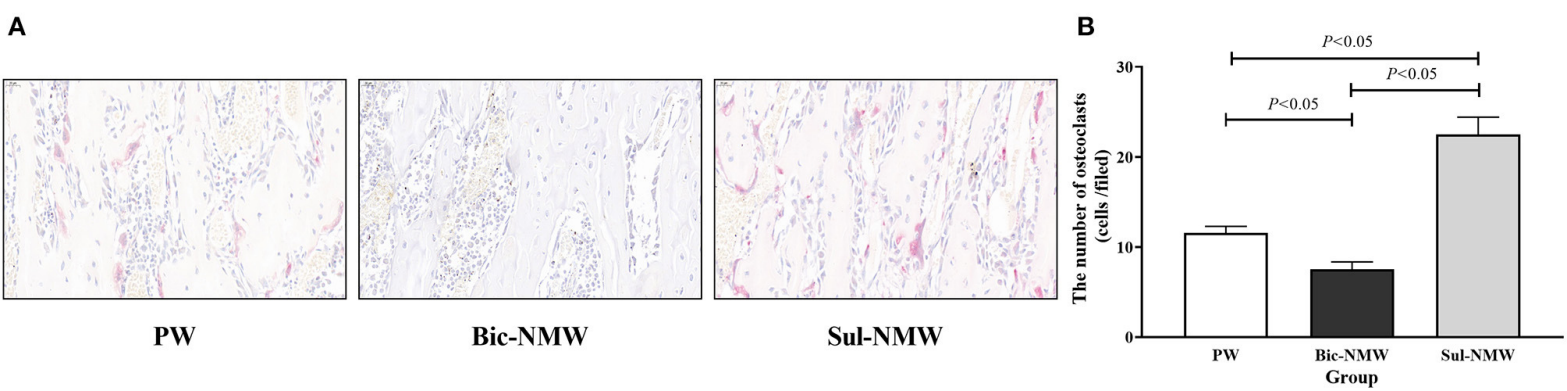
Femoral osteoclasts of young rats with the acid load (16th week). **(A)** Immunostaining of osteoclasts with TRAP (40 ×), scale bar = 20 μm. **(B)** The number of osteoclasts. The values are presented as means with error bars indicating SEM; *n* = 10 rats/group. PW, the purified water group; Bic-NMW, the bicarbonate-rich mineral water group; Sul-NMW, the sulfate-rich mineral water group.

Compared with the PW group, the Bic-NMW group showed a larger cortical bone area (*P* < 0.05, Figures 4A,B). The two mineral water groups had a larger cortical trabecular area, smaller cortical marrow cavity area, and higher growth cartilage width (*P* < 0.05, Figures 4C–E). The Sul-NMW group showed a significantly higher hypertrophic cartage width than the PW group (*P* < 0.05, Figure 4F). There was no significant difference in proliferative zone width, cancellous bone area, cancellous trabecular bone area, and cancellous marrow cavity area among the three groups (*P* ≥ 0.05, Figure 4G, Supplementary Figures 6A–C). The osteoclasts were significantly increased in the Sul-NMW group than that in the PW group and the Bic-NMW group, and were decreased in the Bic-NMW group than that in the PW group (*P* < 0.05, Figures 5A,B).

### Bone Biomechanical Properties After the Acid Load

Compared with the PW group, the Bic-NMW group showed a significantly higher level of ultimate stress (*P* < 0.05, Table 2), and the Sul-NMW group showed a significantly lower maximum deflection and ultimate strain (*P* < 0.05, Table 2), indicating that the Bic-NMW group had the better inner strength and the Sul-NMW group had the better toughness.

**Table 2:** Biomechanical properties of femora of rats with acidosis determined by the Three-Point Bending test.

	**PW**	**Bic-NMW**	**Sul-NMW**
Maximum load (*N*)	118.41 ± 5.02	138.89 ± 6.64	119.45 ± 10.90
Maximum deflection (mm/mm)	1.19 ± 0.10	1.04 ± 0.10	0.87 ± 0.08^a^
Ultimate stress (MPa)	29.32 ± 1.10	35.12 ± 1.69^a^	30.42 ± 2.77
Ultimate strain (mm/mm)	1.38 ± 0.12	1.11 ± 0.12	0.98 ± 0.11^a^

a *P < 0.05 compared with the purified water group*.

*PW, the rats drank the purified water; Bic-NMW, the rats drank the bicarbonate-rich mineral water; Sul-NMW, the rats drank sulfate-rich mineral water*.

*Values are mean ± SEM, n = 9*.

## Discussion

The beneficial effects of drinking mineral water on bone health have been reported by many studies (13, 15, 17, 22). However, whether drinking natural mineral water can prevent bone damage resulting from metabolic acidosis has not been reported. This study successfully established a rat model of metabolic acidosis with NH_4_Cl and assessed the effects of drinking natural mineral water on bone health in rats with acidosis. Our results showed that drinking mineral water, whether alkaline (bicarbonate-rich) or acidic (sulfate-rich) mineral water was beneficial to bone health under acid load.

Although it remains controversial whether the blood pH is altered significantly in rats under acid load, the arterial blood pH (<7.3) is still considered a standard for metabolic acidosis in rats (8, 26). In this study, after treating rats with NH_4_Cl, the blood pH values of all groups were lower than 7.30, and BE, which directly reflected the blood's acid-base buffering capacity, was negative, revealing that these rats endured metabolic acidosis. Drinking mineral water (both Bic-NMW and Sul-NMW) decreased the ${\text{NH}}_{4}^{+}$, TA, and NAE in urine but did not affect the PCO_2_ and ${\text{HCO}}_{3}^{-}$ in blood in rats with acidosis. Rats drinking bicarbonate-rich mineral water had negative NAE and significantly higher urine ${\text{HCO}}_{3}^{-}$ compared with those drinking sulfate-rich mineral water. We conclude that minerals in drinking water can moderate the renal burden for compensating acidosis. However, only when there is enough bicarbonate in mineral water, it can enhance acid buffering capacity (34, 35). This benefit might reduce the need for calcium to buffer the hydrogen ion in blood during renal compensation of acidosis (23, 36), thereby moderating bone resorption induced by renal compensation of acidosis, which is shown by the results in this study that drinking bicarbonate-rich natural mineral water reduced the serum calcium ion and increased vitamin D when compared with drinking sulfate-rich natural mineral water, and decreased blood total calcium and increased serum calcitonin when compared with drinking purified water.

We did not observe alterations in bone microstructure in different drinking water groups before metabolic acidosis, and our previous study suggested that drinking low mineral water impairs bone quality in multi-generation (19). Considering that the consumption of water has been relatively fixed in people for years (11, 13, 15), we speculate that the impact of drinking water on bones takes a much longer time. Thus, the alterations in bone microstructural parameters are more like acute acid load by drinking water. In our study, rats drinking bicarbonate-rich mineral water or sulfate-rich mineral water had higher BMD, higher growth cartilage width, and less marrow cavity area than those drinking purified water. BMD is a vital bone prediction factor for bone strength and fracture (37–39). This finding indicated that drinking natural mineral water can maintain bone mineral content and protect the bone structure in rats with acidosis (22, 40–43).

Meanwhile, rats drinking bicarbonate-rich water had greater BV/TV than those drinking purified water, and had lower BS/BV and higher trabecular thickness than those drinking sulfate-rich natural mineral water. The BV/TV and trabecular thickness are indicators good for bone quality, and BS/BV is a negative indicator for bone (37, 39), suggesting that drinking bicarbonate-rich water may have more benefits on bone microstructure.

In this study, the bone formation marker PINP was not different in the three groups, implying that drinking natural mineral water, no matter sulfate-rich or bicarbonate-rich natural mineral water, cannot promote bone formation compared with drinking purified water. Serum CTx and femoral osteoclasts were lower in rats drinking bicarbonate-rich natural mineral water and higher in rats drinking sulfate-rich natural mineral water than those drinking purified water. CTx is the bone resorption biomarker. Osteoclasts are responsible for bone resorption. We speculate that drinking bicarbonate-rich natural mineral water rich in minerals and alkalis can meliorate the bone resorption induced by acidosis, and that drinking sulfate-rich natural mineral water cannot prevent bone resorption. Our study showed a higher serum BALP concentration after drinking sulfate-rich natural mineral water than drinking purified water and bicarbonate-rich natural mineral water. BALP is secreted by osteoblasts and can enhance extracellular mineralization by hydrolyzing pyrophosphate to inorganic phosphate (44). We assumed that drinking sulfate-rich natural mineral water also exerts a protective effect on bone microstructure. It can maintain bone mineral content by promoting bone mineralization due to increased calcium intake (13, 15, 17).

The higher bone mineralization but deterioration of bone microstructure may increase the brittleness of bone, supporting that drinking sulfate-rich natural mineral water decreased maximum deflection and ultimate strain, the markers of toughness, compared with drinking purified water. Furthermore, drinking bicarbonate-rich natural mineral water increased ultimate stress compared with drinking purified water. Ultimate stress is an indicator of biomechanics and is associated with bone remodeling and bone strength (31). It could be reasonably assumed that it needs not only minerals but also bicarbonate in drinking water to protect biomechanical properties under acidosis.

However, this study has several limitations. First, we could not accurately compute the dosage of NH_4_Cl administered. The results of blood pH showed that we might excessively administrate NH_4_Cl and induce the acidosis too much. Consequently, we could not assess the moderate effect of mineral water on acidosis by evaluating blood pH groups. Second, the NH_4_Cl was administered in water, which might change the alkaline and acidic ions in water and affect the results. Third, we assessed the effects on bone after acid load for only one time point, and bone biomechanical data before acidosis was not tested. It did not fully reflect the effect of drinking mineral water. Fourth, we did not analyze the irons in urine besides calcium and magnesium. Therefore, we could not conclude how drinking mineral water, especially the sulfate-rich natural mineral water, buffered the acid excretion from the kidney and moderated the renal burden for compensating acidosis.

In summary, this study demonstrates that drinking water rich in bicarbonate and minerals may neutralize the acid from NH_4_Cl, buffer acid-base balance, regulate the body's calcium metabolism, and maintain bone microstructure and bone biomechanical properties under acidosis. Therefore, we conclude that minerals and bicarbonate in drinking water can reduce acid load and benefit bone health under acidosis.

## Data Availability Statement

The original contributions presented in the study are included in the article/Supplementary Material, further inquiries can be directed to the corresponding author/s.

## Ethics Statement

The animal study was reviewed and approved by Institutional Animal Use and Care Committee of Army Medical University (Chongqing, China).

## Author Contributions

WS and YH conceived and designed the exposure estimates. YT designed and performed the experiment, statistical analyses, and drafted the manuscript. AX and ZQ assisted with the interpretation of the data and checked the statistical analyses. HJ performed the potential renal acid load analysis. LW, JX, and YW performed the bone examination and sample analysis. JL and HZ assisted with the animal model building. JW modified the manuscript. All authors read and approved the final manuscript.

## Funding

This work was supported by the Military Major Program of People's Liberation Army of China (Grant No. AWS18J004), National Key R&D Program of China (2021YFC3100100), the National Natural Science Foundation of China (Grant Nos. 81803195, 81402647, and 81273029), the Project of Chongqing Municipal Health Bureau (Grant No. 2012-2-447), and the Natural Science Foundation of Chongqing (Grant No. cstc2014jcyjA00049).

## Conflict of Interest

The authors declare that the research was conducted in the absence of any commercial or financial relationships that could be construed as a potential conflict of interest.

## Publisher's Note

All claims expressed in this article are solely those of the authors and do not necessarily represent those of their affiliated organizations, or those of the publisher, the editors and the reviewers. Any product that may be evaluated in this article, or claim that may be made by its manufacturer, is not guaranteed or endorsed by the publisher.

## Abbreviations

25 (OH) vitamin D: 25-dihydroxyvitamin D
ALB: albumin
BALP: bone alkaline phosphatase
BE: base-excess
Bic-NMW: bicarbonate-rich natural mineral water
BMD: bone mineral density
BS/BV: bone surface/bone volume
BV/TV: bone volume/total volume
Ca: calcium
CT: calcitonin
Cl^−^: chloride
CTX: crosslinked C-telopeptide of type I collagen
H^+^: hydrogen ions
${\text{HCO}}_{3}^{-}$: bicarbonate
H_2_SiO_3_: metasilicate acid
K: potassium
Mg: magnesium
micro-CT: three-dimensional microcomputed tomography system
Na: sodium
NAE: net acid excretion
${\text{NH}}_{4}^{+}$: ammonium ion
P: phosphorus
PCO_2_: partial pressure of carbon dioxide
PINP: procollagen I N-terminal propeptide
PRAL: potential renal acid load
PTH: parathyroid hormone
PW: purified water
${\text{SO}}_{4}^{2-}$: sulfate
Sul-NMW: sulfate-rich natural mineral water
TA: titrated acid
Tb. N: trabecular bone number
Tb.Sp: trabecular separation
Tb.Th: trabecular thickness
TDS: total dissolved solids
TH: total hardness
TRAP: tartrate-resistant acid phosphatase.
