# Supplementary material for: Drinking Natural Mineral Water Maintains Bone Health in Young Rats With Metabolic Acidosis
Source: Front Nutr. 2022 Mar 21;9:813202. doi: 10.3389/fnut.2022.813202 (PMC8979287; doi:10.3389/fnut.2022.813202)
Supplement: Supplementary file 1 [file Data_Sheet_1.docx]

**Supplementary data**

**
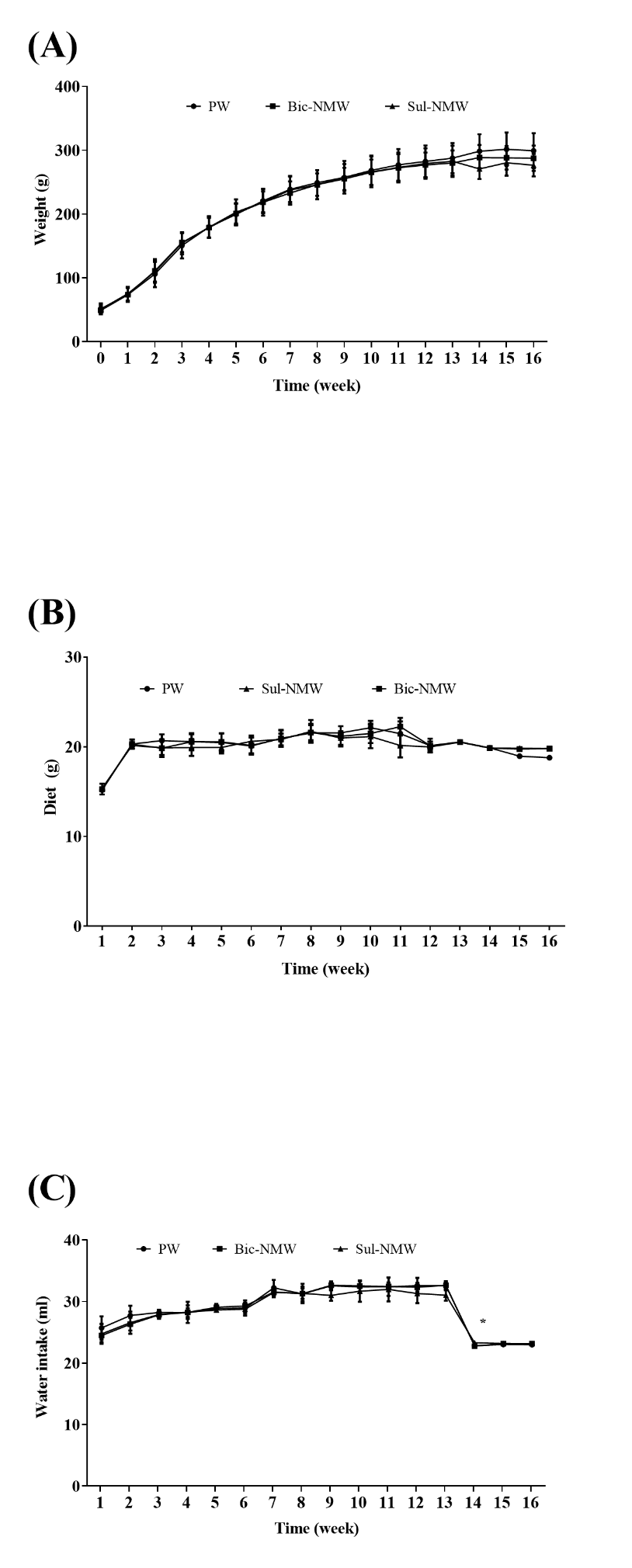
**

**Supplemental Figure 1.** **The body weight (A), diet intake (B), and water intake (C) of the young rats.**

NH_4_Cl was administered through drinking water from the 14^th^ to the 16^th^ week.

The values are presented as means with error bars indicating SEM; n=20 before the 13^th^ week and n=10 after the 14^th^ week.

Abbreviations: PW, the purified water group; Bic-NMW, the bicarbonate-rich mineral water group; Sul-NMW, the sulfate-rich mineral water group.

∗ A significant difference between the 13^th^ and the 14^th^ weeks in each group (*P* <0.05).

**Supplementary data**

**
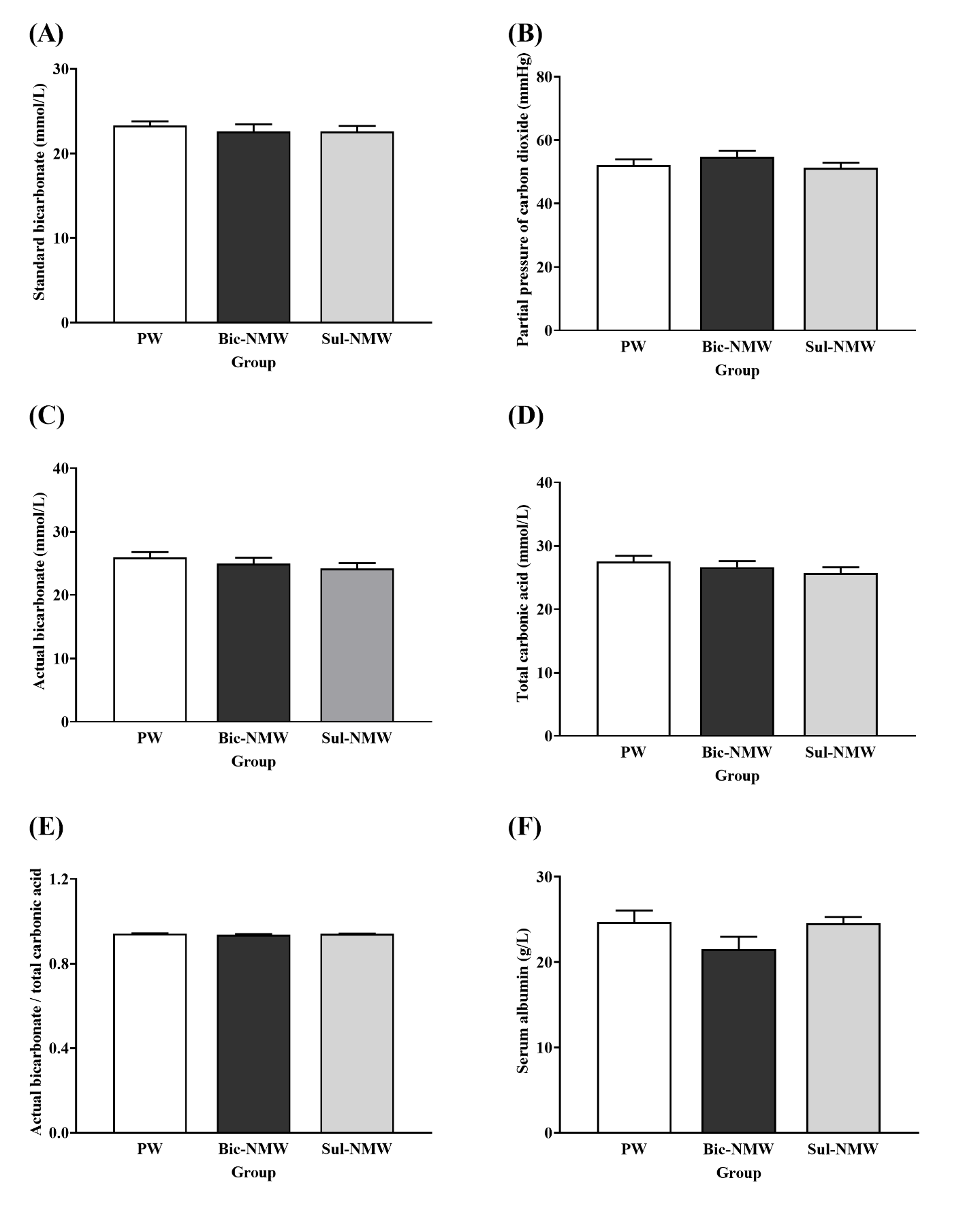
**

**Supplemental Figure 2. The arterial blood acid-base status (A-E) and serum albumin concentration (F) of young rats with the acid load (16^th^ week).**

(A) standard bicarbonate concentration. (B) partial pressure of carbon dioxide. (C) actual bicarbonate concentration. (D) total carbonic acid concentration. (E) the ratio of bicarbonate to total carbonic acid. (F) serum albumin concentration.

The values are presented as means with error bars indicating SEM; n=10.

Abbreviations: PW: the purified water group; Bic-NMW: the bicarbonate-rich mineral water group; Sul-NMW: the sulfate-rich mineral water group.

**Supplementary data**

**
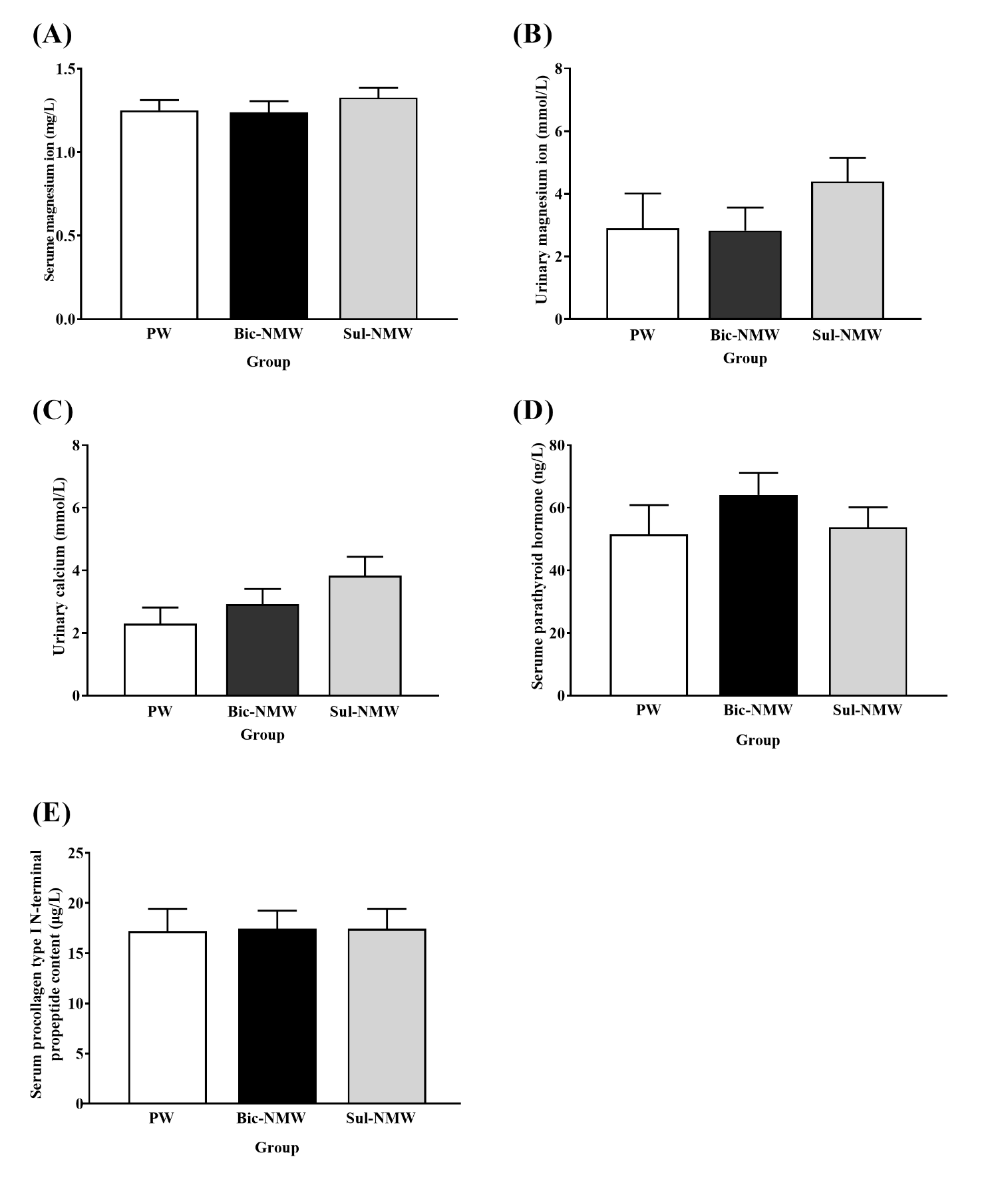
**

**Supplemental Figure 3. The magnesium concentration in serum and urine (A, B), calcium concentration in urine (C), and bone modeling markers serum parathyroid hormone concentration (D) and serum procollagen type I N-terminal propeptide concentration (E) of young rats with the acid load (16^th^ week).**

Serum magnesium concentration (A), urine magnesium concentration (B), urine calcium concentration (C), serum parathyroid hormone concentration (D), and serum procollagen type I N-terminal propeptide concentration (E) of rats with acidosis.

The values are presented as means with error bars indicating SEM; n = 10.

Abbreviations: PW: the purified water group; Bic-NMW: the bicarbonate-rich mineral water group; Sul-NMW: the sulfate-rich mineral water group.

**Supplementary data**

**
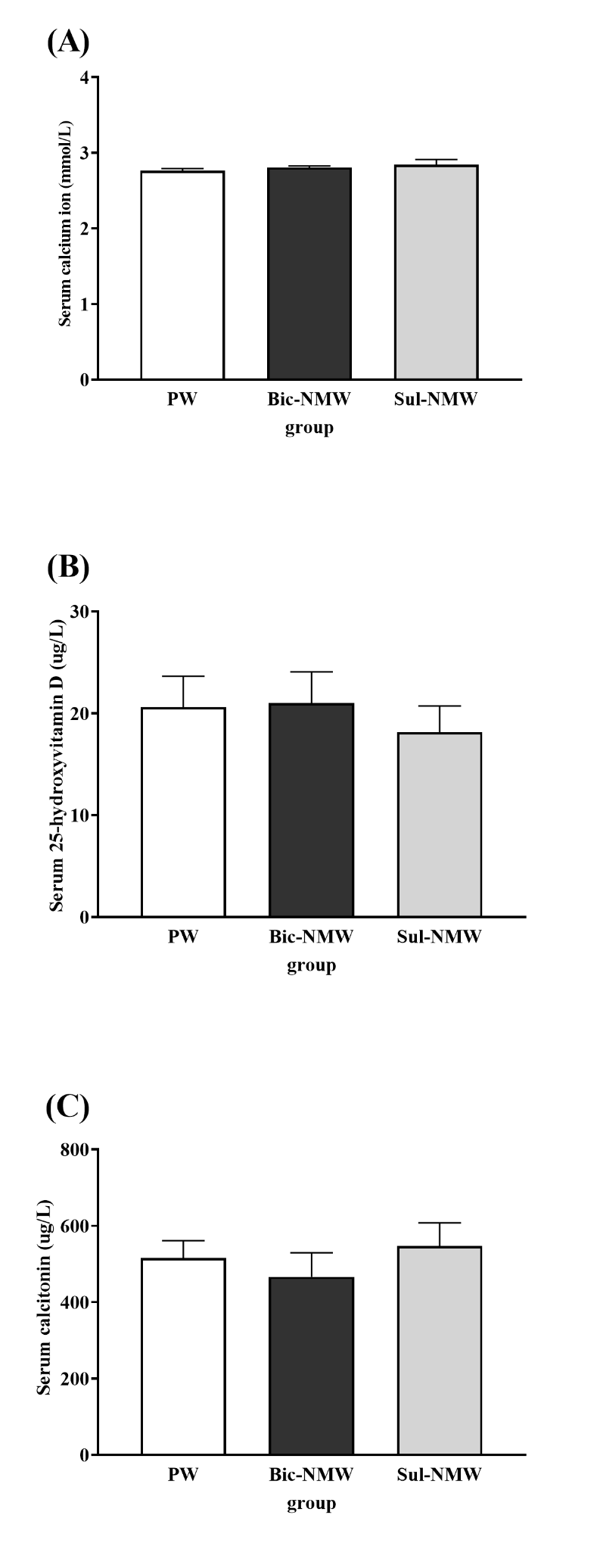
**

**Supplemental Figure 4. Serum calcium and calcium regulatory hormones concentration (A-C) of young rats before the acid load (13^th^ week).**

(A) serum calcium ion concentration. (B) serum 25-hydroxyvitamin D concentration. (C) serum calcitonin concentration.

The values are presented as means with error bars indicating SEM; n=10.

Abbreviations: PW: the purified water group; Bic-NMW: the bicarbonate-rich mineral water group; Sul-NMW: the sulfate-rich mineral water group.

**Supplementary data**

**
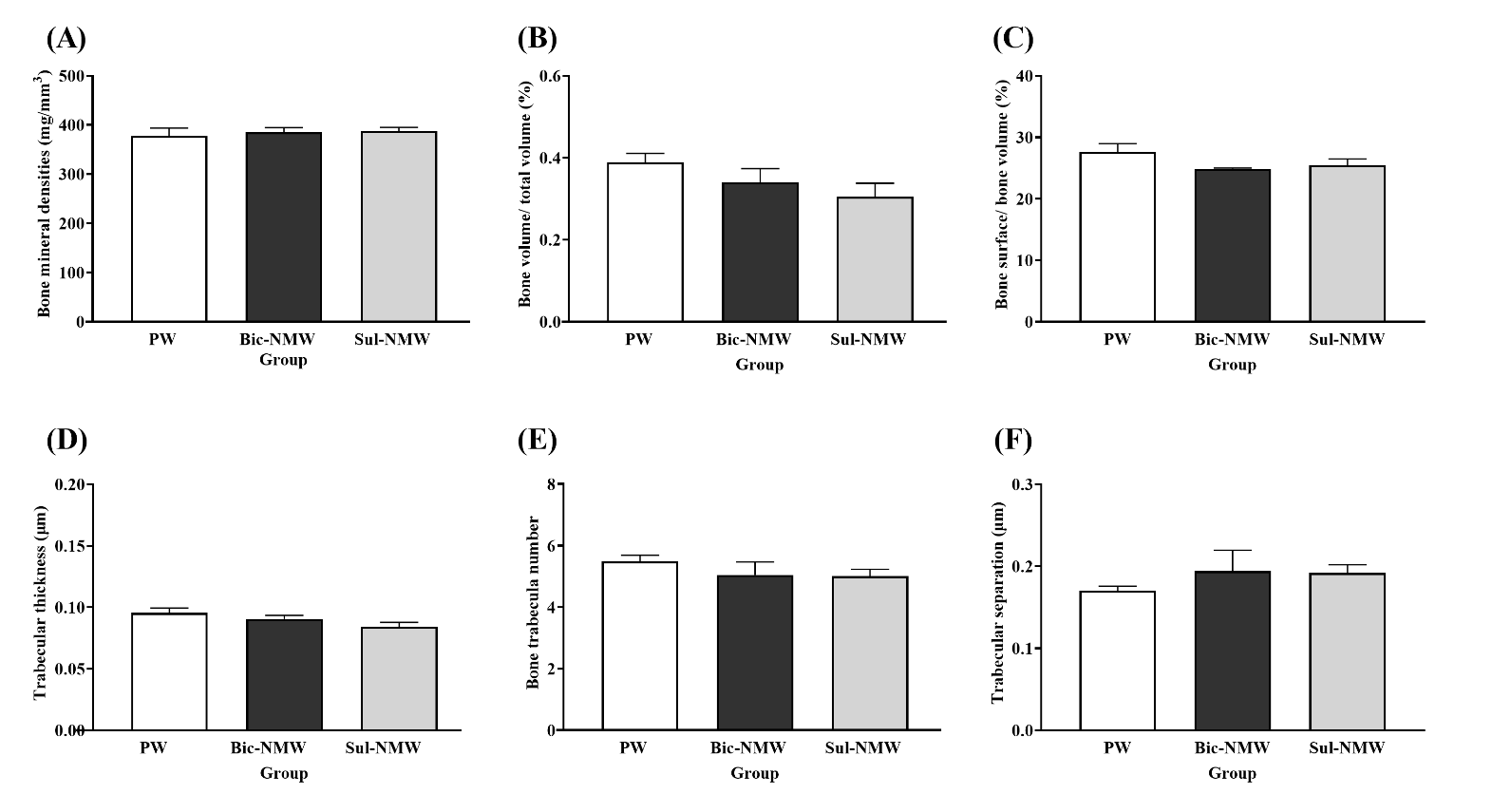
**

**Supplemental Figure 5. Bone microstructure parameters at the 13^th^ week.**

(A) bone mineral density. (B) bone volume/ total volume fraction. (C) bone surface/ bone volume fraction. (D) trabecular thickness. (E) bone trabecula number. (F) trabecular separation.

The values are presented as means with error bars indicating SEM; n = 10.

Abbreviations: PW: the purified water group; Bic-NMW: the bicarbonate-rich mineral water group; Sul-NMW: the sulfate-rich mineral water group.

**Supplementary data**

**
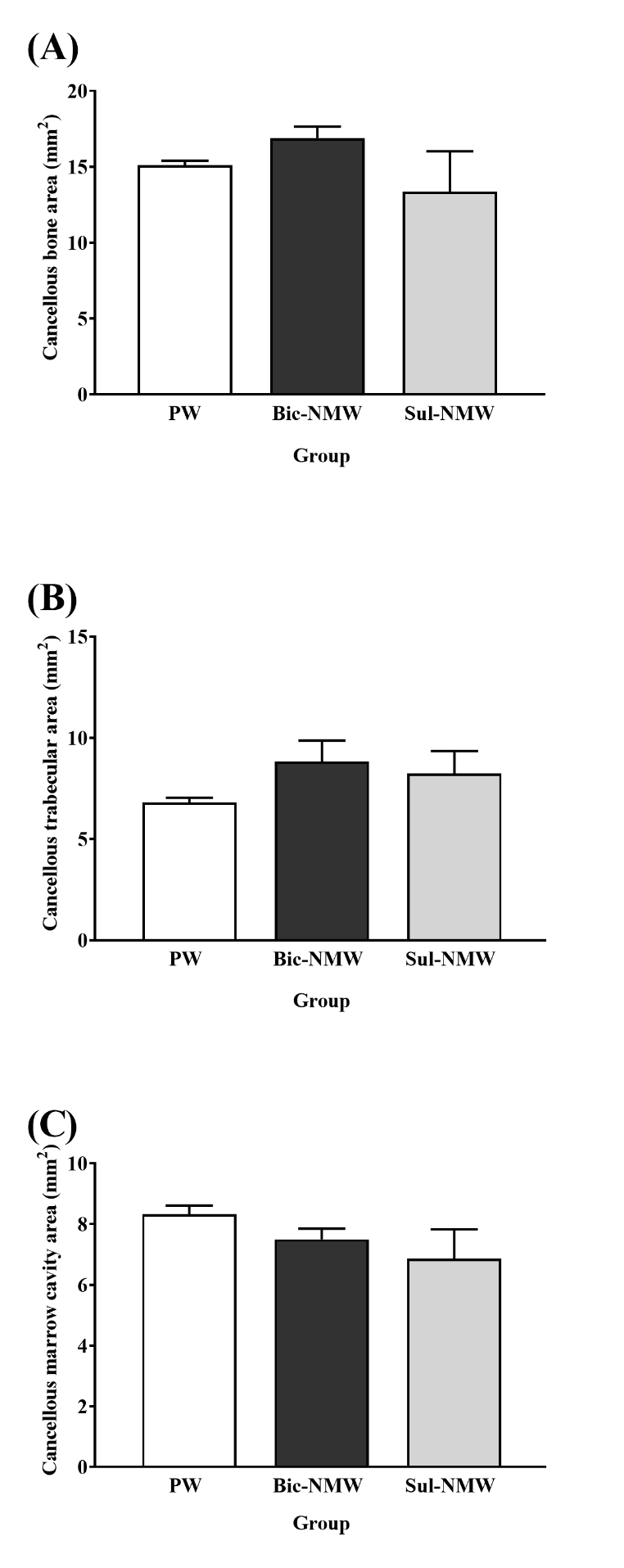
**

**Supplemental Figure 6. Histomorphometric characteristics of the cancellous bone of young rats with the acid load (16^th^ week).**

Femoral cancellous bone area (A), cancellous trabecular bone area (B), and cancellous marrow cavity area (C) of rats in the three groups after the acid load was induced.

The values are presented as means with error bars indicating SEM; n = 10.

Abbreviations: PW: the purified water group; Bic-NMW: the bicarbonate-rich mineral water group; Sul-NMW: the sulfate-rich mineral water group.
